# Особенности биоэнергетического метаболизма в физиологических и патологических условиях: фокус на онкогенез

**DOI:** 10.14341/probl13648

**Published:** 2026-01-18

**Authors:** А. С. Жданова, Ж. Е. Белая, Г. А. Мельниченко

**Affiliations:** Национальный медицинский исследовательский центр эндокринологии им. академика И.И. ДедоваРоссия; Endocrinology Research CentreRussian Federation

**Keywords:** энергообмен, клетка, гликолиз, окислительное фосфорилирование, опухоль, нейроэндокринные образования, диагностика, energy metabolism, cell, glycolysis, oxidative phosphorylation, tumor, neuroendocrine neoplasms, diagnostics

## Abstract

В основе жизнедеятельности каждой клетки организма лежит энергетический обмен, необходимый для реализации физиологических потребностей в норме и при патологии. Важнейшими путями синтеза аденозинтрифосфата являются гликолиз, цикл трикарбоновых кислот и окислительное фосфорилирование. В качестве субстрата для получения энергии могут быть использованы глюкоза, свободные жирные кислоты и аминокислоты. При развитии заболевания в клетках происходит перепрограммирование с возможностью переключения между путями получения энергии и выбором ее источников, формируя специфический метаболический фенотип, обеспечивающий выживание клеток и формирование клинических характеристик болезни. Наличие информации о патофизиологических изменениях на уровне метаболизма клеток представляет научно-практический интерес в отношении разработки методов для точной диагностики и выбора персонализированной тактики в каждом конкретном случае. В представленном обзоре охарактеризованы особенности энергетического метаболизма в норме и в опухолевых клетках. Собрана информация о современных методах оценки уровня энергообмена в организме.

## Abstract (en)

The basis of the vital activity of each cell of the body is energy metabolism, necessary for the implementation of physiological needs in norm and pathology. The most important pathways for the synthesis of adenosine triphosphate are glycolysis, the tricarboxylic acid cycle and oxidative phosphorylation. Glucose, free fatty acids and amino acids can be used as a substrate for obtaining energy. As the disease develops, reprogramming occurs in cells with the ability to switch between energy pathways and the choice of its sources, forming a specific metabolic phenotype that ensures cell survival and the formation of clinical characteristics of the disease. The availability of information on pathophysiological changes at the level of cell metabolism is of scientific and practical interest in relation to the development of methods for accurate diagnosis and the choice of personalized tactics in each specific case. This review describes the characteristics of energy metabolism in normal and tumor cells. It also provides information on modern methods for assessing energy metabolism in the body.

## ВВЕДЕНИЕ

Функционирование каждой живой клетки человека сопряжено и зависит от наличия энергии для обеспечения процессов жизнедеятельности, таких как рост, дифференцировка, поддержание целостной структуры, реагирование на внутри- и внеклеточные факторы. Процессы энергообеспечения организма представляют собой совокупность сложных биохимических реакций, в результате которых пищевые компоненты трансформируются в молекулу аденозинтрифосфата (АТФ) — главного источника энергии живых клеток. Для сохранения стабильности внутренней среды клетки и обеспечения эффективной работы органов и тканей в организме требуется адекватный контроль за протеканием всех метаболических этапов энергообмена. Перепрограммирование путей синтеза АТФ лежит в основе клеточной дисфункции, повреждения тканей и нарушения системного гомеостаза, что является краеугольным камнем в патогенезе широкого спектра заболеваний, включая эндокринную патологию [1]. При нарушении функции щитовидной железы, сахарном диабете, гиперкортицизме нарушение процессов энергетического обмена в каждой клетке становится ключевым звеном патогенеза заболевания на клеточном уровне [2]. При онкологической патологии также происходит изменение метаболизма опухолевых клеток, которое часто используется в том числе для функциональных методов визуализации [1]. Вместе с тем остаются открытыми множество вопросов относительно изменений энергетического обмена в клетках: механизмы преобразования энергии, регуляторные сигналы, межклеточное взаимодействие при передаче сигналов. Понимание патологических изменений энергообмена в клетках открывает новые стратегические направления для разработки таргетной диагностики, прогнозирования течения болезни и персонализированных терапевтических вмешательств, нацеленных на коррекцию метаболических путей. В данном обзоре мы акцентируем внимание на изменении метаболического статуса клеток в опухолевой ткани, в том числе при нейроэндокринных образованиях.

## ЭНЕРГЕТИЧЕСКИЙ МЕТАБОЛИЗМ В ФИЗИОЛОГИИ

Биоэнергетический аппарат клетки представлен гликолитическим путем в цитоплазме и митохондриальным компартментом (включает цикл трикарбоновых кислот или «цикл Кребса» и окислительное фосфорилирование), конечным продуктом которых является обеспечение клетки аденозин-5’-трифосфатом (АТФ) [3].

В ходе гликолиза («glykys» + «lysis» = «разделение сладкого»), также известного как путь Эмбдена-Мейергофа-Парнаса в честь его первооткрывателей, в клетке происходит образование пирувата или пировиноградной кислоты, что обеспечивает клетку 2 молекулами АТФ [4].

Выделяют несколько важных аспектов гликолитического пути получения энергии: обратимость и возможность реакций идти в противоположном направлении (например, глюконеогенез), взаимосвязь гликолиза с пентозофосфатным путем, липогенезом, синтезом нуклеотидов и гликогенезом; ввиду быстроты реакции гликолиза данный путь эффективен для клеток с высокой скоростью пролиферации или клеток, подвергаемых экстремальным нагрузкам [3].

Кроме того, гликолиз используется в качестве основного способа выработки АТФ в условиях недостатка кислорода или в клетках, не имеющих в своем составе митохондрий, — в эритроцитах [1].

В случае аэробных условий конечный продукт гликолиза преобразуется в ацетил-коэнзим А с помощью пируватдегидрогеназы, что активирует цикл трикарбоновых кислот с образованием 2 молекул АТФ, после чего запускается процесс окислительного фосфорилирования с образованием 34 молекул АТФ. Конечным результатом окисления 1 молекулы глюкозы является синтез 38 молекул АТФ (рис. 1) [4].

**Figure:**
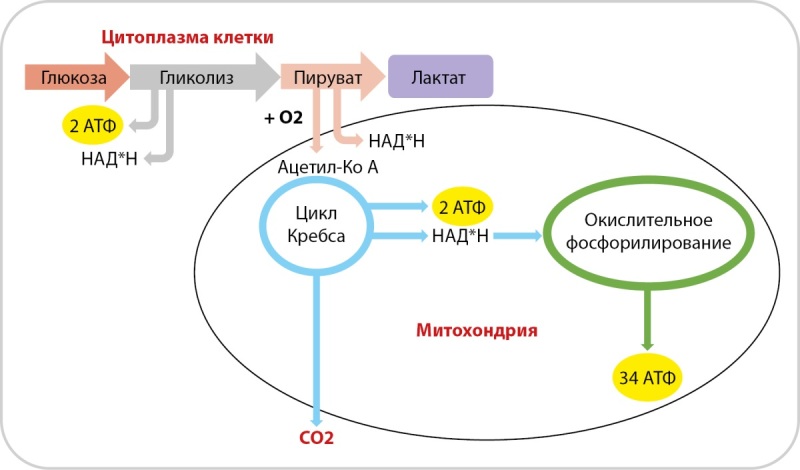
Рисунок 1. Физиология процесса синтеза аденозинтрифосфата. Примечание. В ходе гликолиза в клетке происходит синтез 2 молекул АТФ с образованием пирувата. В аэробных условиях из пирувата синтезируется ацетил-коэнзим А с выработкой 2 молекул АТФ и последующим процессом окислительного фосфорилирования с образованием 34 молекул АТФ. В анаэробных условиях пируват превращается в лактат. АТФ-аденозинтрифосфат; НАД*Н — никотинамидадениндинуклеотид плюс водород.

В качестве субстрата для выработки энергии митохондрии в процессе цикла Кребса и окислительного фосфорилирования могут использовать глюкозу, глутамин, жирные кислоты, а также аминокислоты [4].

При завершении цикла жизнедеятельности процесс апоптоза клетки также является энергозатратным (расщепление ДНК, сжатие цитоплазмы), для чего клетка активирует гликолитический путь получения энергии, а митохондрии в свою очередь участвуют в регуляции апоптоза [1]. В отдельных ситуациях клетки используют альтернативный путь получения энергии — аутофагию (расщепление белков, органелл и других крупных молекул при помощи лизосом) [1].

В анаэробных условиях происходит преобразование пирувата в лактат с помощью лактатдегидрогеназы [4]. Однако лактат выступает в роли энергетического метаболита, а не является конечным продуктом жизнедеятельности клетки, что впервые проявилось в цикле Кори и стало прорывом в 1929 г. Позже было обнаружено, что лактат перерабатывается и накапливается в виде гликогена в печени, мышцах, почках и головном мозге [5]. В ряде случаев возможно превращение глюкозы в лактат и в аэробных условиях, так называемый «аэробный гликолиз», или «эффект Варбурга» [4]. Еще в 1923 г. Отто Варбург обнаружил, что многие типы раковых клеток постоянно используют гликолитическую ферментацию независимо от концентрации кислорода: то есть клетки вырабатывают АТФ в основном посредством окислительного фосфорилирования в аэробных условиях и переключаются на гликолитическое брожение при гипоксии [3]. При этом в высокодифференцированных клетках этот процесс составляет около 1%, а в быстрорастущих низкодифференцированных клетках — 20% [3].

## ПЕРЕПРОГРАММИРОВАНИЕ ЭНЕРГООБМЕНА ПРИ ОПУХОЛЯХ

Метаболические изменения опухолевых клеток представляют собой трансформацию биохимических путей анаболизма и катаболизма, направленную на поддержание их пролиферации и выживания в условиях специфического микроокружения. Механизмы адаптации или так называемый «метаболический фенотип», обеспечивают раковые клетки необходимым энергетическим и биосинтетическим ресурсом для быстрого роста и метастазирования новообразования, а также влияют на процессы клеточной дифференцировки и взаимодействие с окружающими тканями, что способствует долгосрочной персистенции опухоли [6].

Pavlova N.N. и Thompson C.B. выделили основные характеристики метаболизма опухолевых клеток, объясняющих их выживание: 1) нарушение регуляции поглощения глюкозы и аминокислот; 2) использование промежуточных продуктов гликолиза/цикла трикарбоновых кислот для биосинтеза и производства восстановленного никотинамидадениндинуклеотида (НАДН); 3) использование оппортунистических способов получения питательных веществ (например, аутофагия внутриклеточных белков или целых внутриклеточных структур, таких как рибосомы, митохондрии и части эндоплазматического ретикулума); 4) повышенная потребность в азоте для синтеза макромолекул; 5) сложные взаимодействия между метаболизмом и микроокружением опухоли; 6) изменения в регуляции генов, зависящих от метаболитов; 7) потребность в защите от окислительного стресса; 8) гетерогенность метаболических адаптаций разных типов опухолей [7].

Ключевой патофизиологической характеристикой многих солидных новообразований является развитие гипоксии с уровнем оксигенации на 1–2% ниже, чем в здоровых тканях. Патогенез опухолевой гипоксии связан с тремя основными механизмами: аномальной архитектоникой сосудистой сети, увеличенным расстоянием между клетками и сосудами, ограничивающими диффузию кислорода, и снижением кислородтранспортной функции крови при анемии. Снижение концентрации кислорода в опухолевых клетках активирует сигнальные пути, регулирующие ключевые клеточные процессы: пролиферацию, апоптоз, воспалительный ответ, миграцию, выживаемость и метаболическую адаптацию [8].

Фактор транскрипции HIF1 (Hypoxia-inducible factors — фактор, индуцирующий гипоксию) оказывает ключевое влияние на изменение метаболизма глюкозы при развитии гипоксии. Он индуцирует митохондриальную деградацию посредством BNIP3-зависимой (BCL2/adenovirus E1B 19kDa interacting protein3) аутофагии и подавляет биогенез митохондрий через ингибирование транскрипционного фактора MYC (avian myelocytomatosis viral oncogene homolog — ген, кодирующий ядерный фосфопротеин) [8].

В 1956 г. Варбург предположил, что причиной снижения окислительного фосфорилирования в опухолевых клетках является повреждение митохондрий, однако позже эта теория была опровергнута, поскольку аэробный гликолиз в ряде случаев может встречаться и в нормальных пролиферирующих клетках [9].

Митохондриальная дисфункция характерна лишь для ограниченного числа опухолевых линий. К числу таких нарушений относятся: сниженная экспрессия транспортных белков и ферментов окислительно-восстановительных реакций, укорочение цикла Кребса, уменьшение числа митохондрий, повышенная экспрессия ингибиторов АТФ-синтазы в митохондриях, а также повышенная чувствительность митохондриальной ДНК к оксидативному повреждению [8].

Объяснение выбора опухолевыми клетками именно гликолитического пути синтеза энергии складывается из нескольких факторов [10].

Во-первых, несмотря на низкую продукцию АТФ при помощи гликолиза, скорость выработки АТФ в цитоплазме 100-кратно превышает скорость синтеза в митохондриях, в связи с чем клетка получает АТФ в процессе гликолиза за единицу времени больше, чем при окислительном фосфорилировании [10].

Во-вторых, в ходе гликолиза происходит накопление промежуточных продуктов, которые необходимы для биосинтеза макромолекул, таких как липиды, нуклеиновые кислоты и белки, тем самым поддерживая быстрый рост и пролиферацию опухоли [1].

В-третьих, в условиях активации гликолиза наблюдается редукция синтеза свободных радикалов, что минимизирует повреждение ДНК и других клеточных компонентов, обеспечивая ускользание от апоптотической гибели опухолевых клеток [8].

Немаловажную роль для создания условий функционирования опухолевых клеток играет лактат. Под действием лактатдегидрогеназы A из пирувата образуется молочная кислота, которая при помощи карбоксильного транспортера высвобождается за пределы клетки и модулирует кислотно-щелочной баланс, снижая pH среды опухолевых клеток [11].

Молочная кислота потенцирует супрессию врожденного иммунного ответа через ингибирование RIG-I-зависимой (retinoic acid-inducible gene I = ген I, индуцируемый ретиноевой кислотой) продукции интерферона I типа (IFN). Сдвиг pH в кислую сторону в опухолевом микроокружении стимулирует экзоцитоз лизосомальных гидролаз и активирует внутриклеточные сигнальные пути, способствуя инвазии и метастазированию [11].

Усиление процессов аэробного гликолиза в опухолевых клетках приводит к ограничению поступления пирувата в цикл трикарбоновых кислот, в связи с чем активация глутаминолиза пополняет цикл Кребса промежуточными продуктами из глутамина и обеспечивает энергией при анаплерозе (пополнение, возмещение потраченных частей) (рис. 2) [12].

**Figure:**
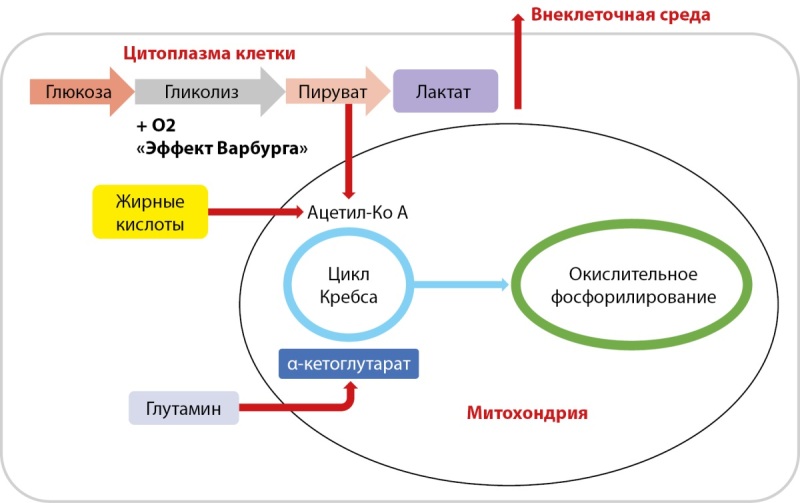
Рисунок 2. Изменение энергообмена в опухолевой ткани. Примечание. Составляющие метаболической адаптации опухолевых клеток: 1) активация аэробного гликолиза — «Эффект Варбурга»; 2) снижение поступления пирувата в цикл трикарбоновых кислот; 3) активация глутаминолиза с пополнением цикл Кребса промежуточными продуктами (α-кетоглутарат); 4) увеличение продукции лактата с поступлением его во внеклеточную среду и снижением pH среды опухолевых клеток; 5) активация фосфорилирования жирных кислот с образованием Ацетил-КоА.

Считавшийся ранее заменимой аминокислотой глутамин был реклассифицирован после открытия Lacey J.M. и др. в 1990 г. Авторы продемонстрировали, что в состояниях катаболизма эндогенный синтез глутамина становится недостаточным для покрытия метаболических потребностей, что послужило основанием для отнесения его к категории условно незаменимых аминокислот [12].

После первоначального наблюдения Eagle и коллег, выявившего исключительно высокую скорость потребления глутамина опухолевыми клетками HeLa (на порядки превышающую таковую для других аминокислот), повышенный метаболизм глутамина стал ассоциироваться с процессами опухолевого роста, инвазии и метастазирования при различных злокачественных новообразованиях, включая рак яичников, молочной железы и поджелудочной железы [12].

После транспорта в клетку глутамин в митохондриях подвергается ферментативному дезаминированию под действием глутаминазы с образованием глутамата. Последующий катаболизм глутамата, опосредованный глутаматдегидрогеназой, приводит к синтезу α-кетоглутарата — ключевого интермедиата цикла трикарбоновых кислот. Инкорпорация α-кетоглутарата в цикл Кребса обеспечивает генерацию восстановленных коферментов (НАДН, ФАДН — флавинадениндинуклеотид восстановленный), которые используются в процессе окислительного фосфорилирования для синтеза АТФ, тем самым удовлетворяя энергетические потребности клетки [10]. Дополнительно аминокислота может выступать в роли сигнальной молекулы, стимулирующей комплекс 1 мишени рапамицина (mTORC1) и способствуя росту опухолевых клеток [11].

Помимо этого, метаболиты глутамина вносят вклад в синтез жирных кислот в раковых клетках с дисфункцией цикла трикарбоновых кислот. Ключевым посредником в этом процессе выступает цитрат, образующийся посредством восстановительного карбоксилирования (присоединение СО2 к предшественнику оксалоацетату с образованием цитрата). Данный механизм предполагает конверсию α-кетоглутарата в цитрат, катализируемую изоцитратдегидрогеназами. Экспериментальные данные свидетельствуют об активности восстановительного карбоксилирования в условиях гипоксии in vitro, а также подтверждают его существенную роль в обеспечении липогенеза в процессе развития злокачественных новообразований in vivo [12].

Важным отличием метаболизма опухолевых клеток является аномальное фосфорилирование жирных кислот. Жирные кислоты являются не только основными компонентами мембран, но и источником энергии, а также могут выполнять роль вторичных посредников передачи сигналов в быстро пролиферирующих опухолевых клетках. Когда потребность клеток в энергии возрастает, жирные кислоты транспортируются в митохондрии для β-окисления, где они преобразуются в ацетил-КоА и впоследствии вступают в цикл трикарбоновых кислот для выработки АТФ [1][10].

Нарушение липидного обмена выступает важным фактором риска развития нейроэндокринных образований, и его регулирование может быть потенциальной терапевтической мишенью.

Tianshun F. и соавт. опубликовали результаты работы по анализу данных 146 пациентов с нефункционирующими нейроэндокринными опухолями гипофиза (NF-PitNET). Согласно клиническим данным и биоинформатическому анализу, гены, связанные с метаболизмом холестерина, могут способствовать обильной инфильтрации иммунных клеток при гормонально-неактивных-PitNET и инвазии кавернозных синусов гормонально-неактивных-PitNET через сигнальный путь mTOR. Данное исследование открывает новые перспективы для изучения патогенеза инвазии кавернозных синусов NF-PitNET [13].

Одним из ярких примеров гетерогенности метаболических адаптаций является предпочтение определенного анаплеротического субстрата для цикла трикарбоновых кислот [9].

В опухолевых клетках может преобладать одна из двух противоположных стратегий: 1) восполнение цикла трикарбоновых кислот происходит за счет α-кетоглутарата, образующегося в результате катаболизма глутамина; 2) пул промежуточного продукта цикла трикарбоновых кислот оксалоацетата пополняется за счет карбоксилирования пирувата в реакции, катализируемой пируват-карбоксилазой [9].

Зависимость от конкретного способа получения энергии может указывать на связь между метаболическим характером исходной ткани и возникающими из нее опухолями. Например, опухоли поджелудочной железы и лёгких преимущественно получают АТФ с использованием карбоксилирования пирувата, в то время как колоректальный рак использует глутамин в качестве основного субстрата [9].

Использование селективного воздействия на процессы гликолиза при гликолитическом фенотипе опухоли, влияние на метаболизм глутамина, а также фармакологическое модулирование синтеза жирных кислот становится эффективным способом в терапии онкологических заболеваний.

Перспективной мишенью для действия лекарственных препаратов выступает фермент гликолиза — гексокиназа-2, при блокировании которого наблюдается запуск апоптоза раковых клеток. Такие ингибиторы фермента, как 3-бромпируват, лонидамид, 2-дезоксиглюкоза, способны подавлять гликолиз в опухолевых клетках, дополнительно повышая цитотоксические эффекты противоопухолевых препаратов. Дополнительными точками воздействия являются ферменты лактатдегидрогеназа и пируватдегидрогеназа, воздействуя на которые можно снижать продукцию лактата и пирувата в раковых клетках, влияя на активность их пролиферации [14].

В зависимости от дифференцировки и происхождения опухолевых клеток, а также при наличии секреторной активности гормонов или других медиаторов клеточный метаболизм может принципиально отличаться, что в том числе положено в основу функциональных методов визуализации, в частности основного радиофармпрепарата ¹⁸F-ФДГ (Фтордезоксиглюкоза 18) при позитронно-эмиссионной томографии, совмещенной с компьютерной томографией (ПЭТ-КТ). Принципиально иное изменение обмена энергии происходит в нейроэндокринных опухолях (НЭО), которые нередко обладают способностью секретировать биологически активные соединения.

## ОСОБЕННОСТИ ЭНЕРГООБМЕНА ПРИ НЕЙРОЭНДОКРИННЫХ ОПУХОЛЯХ

Нейроэндокринные опухоли (НЭО) представляют гетерогенную группу неоплазий, происходящих из пептидергических нейронов и диффузной нейроэндокринной системы, диссеминированной в различных органах и тканях. Наиболее частая локализация включает респираторный тракт и желудочно-кишечную систему, тогда как реже поражаются надпочечники, тимус и другие органы [15].

Согласно эпидемиологическим данным, до 0,5% всех впервые диагностированных злокачественных новообразований составляют НЭО [16].

По функциональной активности все нейроэндокринные образования разделяют на функционирующие и нефункционирующие [17].

Нефункционирующие НЭО могут длительно существовать бессимптомно, нередко являясь случайной находкой при обследовании по иному заболеванию, поскольку не имеют специфической симптоматики [17].

Примерно 20% НЭО являются функционирующими и обладают способностью к синтезу и секреции определенных вазоактивных пептидов, аминов, гормонов, приводя к появлению яркой клинической симптоматики, что позволяет врачам заподозрить наличие заболевания и провести диагностическую оценку биомаркеров, характерных для синдромального диагноза по результатам клиники [15][17].

Среди неспецифических биомаркеров, характерных как для нефункционирующих, так и для функционирующих НЭО, выделяют: хромогранин А, панкреатический полипептид (НЭО поджелудочной железы), нейронспецифическая енолаза (НЭО легких и тимуса).

Среди специфических маркеров выделяют: серотонин, 5-гидроксииндолуксусная кислота (для карциноидного синдрома), гастрин (для гастриномы), глюкагон (для глюкагономы), инсулин и С-пептид (для инсулиномы) и др. [17].

Алгоритм выбора тактики ведения пациента зависит от типа и распространенности опухоли, а также от градации потенциала злокачественности НЭО по Grade (G1-3). Определение степени злокачественности для НЭО легких, поджелудочной железы и желудочно-кишечного тракта имеет различия в подходах, однако учитывает уровень пролиферативной активности клеток по митотическому индексу, индексу Ki-67 [17].

Высокодифференцированные НЭО составляют приблизительно 80% в структуре нейроэндокринной опухолевой патологии. Данная категория опухолей характеризуется низким пролиферативным потенциалом, более мягким/условно доброкачественным течением, а прогностические исходы варьируют от благоприятных до умеренных в зависимости от стадии заболевания, первичной локализации, уровня митотической активности [15].

К высокодифференцированным НЭО относят: НЭО пищеварительного тракта (митотический индекс для G1<2, Ki-67≤2%, для G2 митотический индекс =2–20, Ki-67=3–20%), НЭО легких и тимуса (типичный карциноид, низкий митотический индекс <2/10РП31 G1; атипичный карциноид, высокий митотический индекс 2–10/10РП3 G2). Также к этой группе можно отнести: карциноиды различного происхождения, опухоли из хромаффинных клеток (феохромоцитома, параганглиома), медуллярный рак щитовидной железы [18].

Низкодифференцированные нейроэндокринные образования включают градацию G3 с Ki-67>20% или с числом митозов >20 в 10 полях зрения. Низкодифференцированные НЭО в свою очередь характеризуются агрессивным течением, выраженной ядерной атипией, мультифокальными некрозами и высокой частотой метастазирования. К G3 относят мелко- и крупноклеточный нейроэндокринный рак легких [18].

В последние десятилетия наблюдается устойчивая тенденция к улучшению показателей общей выживаемости пациентов с нейроэндокринными новообразованиями. Тем не менее у 12–21% пациентов на момент диагностики заболевания имеются метастазы, что актуализирует необходимость идентификации новых высокоспецифичных биомаркеров, способных обеспечить своевременную диагностику и повышение эффективности радикального лечения [15].

НЭО имеют специфические характеристики, затрудняющие их диагностику с использованием стандартных методов: при НЭО клетки используют преимущественно глутаминолиз в митохондриях вместо гликолиза, в связи с чем не происходит захват ¹⁸F-ФДГ во время проведения исследования ПЭТ-КТ. Высокодифференцированные НЭО имеют низкую гликолитическую активность (незначительное усиление эффекта Варбурга). Клетки активно используют альтернативные субстраты получения энергии (глутамин, жирные кислоты), а также могут иметь слабую экспрессию транспортеров глюкозы GLUT1 и GLUT3 [9].

Таким образом, метаболическая адаптация опухолевых клеток представляет собой сложный процесс в виде переключения между способами синтеза энергии, обеспечивающий адаптивное выживание раковых клеток, что требует применения персонализированных методов диагностики с учетом изменения энергообмена клеток.

## СПОСОБЫ ОПРЕДЕЛЕНИЯ УРОВНЯ ЭНЕРГООБМЕНА КЛЕТКИ И ИХ РОЛЬ В КЛИНИЧЕСКОЙ ПРАКТИКЕ

С целью выявления аномального клеточного метаболизма перспективным направлением становится оценка промежуточных продуктов энергообмена, в частности глюкозы, лактата, НАДН, глутамата и АТФ, непосредственно участвующих в процессах гликолиза и окислительного фосфорилирования [1].

Основные существующие лабораторно-инструментальные способы определения энергообмена в клетках перечислены в таблице 1.

**Table:** Таблица 1. Методы косвенной оценки энергетического метаболизма клеток Примечание: НАД — никотинамидадениндинуклеотид; НАДН-восстановленный НАД; ЛДГ — лактатдегидрогеназа, МРТ — магнитно-резонансная томография, ПЭТ — позитронно-эмиссионная томография, ВЭЖХ — высокоэффективная жидкостная хроматография, ГХ — газовая хроматография, МС — масс-спектрометрия, КЭ-МС — капиллярный электрофорез-масс-спектрометрия, GLUT — глюкозный транспортер (Glucose Transporter).

	Методы, используемые в рутинной практике	Методы, используемые в научных исследованиях
Биохимический анализ	МРТ и ПЭТ/КТ	Спектрометрия	Молекулярная диагностика	Хроматография	Масс-спектрометрия
Определение показателей	Глюкоза (кровь, моча)Липиды (кровь)Лактат (кровь, моча)GLUT (кровь)Пируват (кровь)ЛДГ — кровь, моча, слюна	Глюкоза — МРТ и ПЭТ/КТ (18-FDG)Липиды — МРТЛактат-МР спектроскопияGLUT — ПЭТ/КТПируват- ЯМР-спектроскопияАминокислоты — ЯМР-спектроскопия	Лактат-спектрометрия и колориметрияПируват-колориметрияНАДН и НАД*- колориметрияАминокислоты — спектрометрия и колориметрияЛДГ-колориметрия	Лактат-флуоресцирующий зондGLUT-электрохимический зондНАДН и НАД*-флуоресцирующий зондАТФ — флуоресцирующий зонд	Лактат (слюна, плазма и моча) — ВЭЖХПируват — ВЭЖХНАДН и НАД* — ВЭЖХАминокислоты — ВЭЖХЛипиды-ГХЛДГ-ВЭЖХ, ГХ	НАДН и НАД*-ЖХ и МСАминокислоты — ЖХ и МС, КЭ-МС, ГХ — МСЛипиды-ГХ — МСПируват-ЖХ и МСГлюкоза — ЖХ гидрофильного взаимодействия с тандемной МС

Биохимический анализ в рамках лабораторной диагностики биологических жидкостей (крови, мочи, слюны) используется для определения следующих метаболитов: глюкоза, липиды, лактат, пируват, лактатдегидрогеназа, GLUT. Часть этих технологий используется для рутинной диагностики заболеваний, которые неизбежно приводят к нарушению обмена энергии, в частности при сахарном диабете, а также при различных видах органной недостаточности [1].

Наиболее активно изменения метаболизма клеток используются для функциональных методов визуализации опухолевых клеток или нарушений метаболизма. Функциональная визуализация позволяет неинвазивно охарактеризовать метаболический статус и гетерогенность опухоли на основе анализа интенсивности поглощения радиофармпрепаратов, а также обеспечить более точную прогностическую стратификацию [19].

Стратегия разработки радиофармпрепаратов (РФП) для диагностики НЭО основывалась на трех основных биологических мишенях: сверхэкспрессия опухолеспецифичных мембранных рецепторов, активный транспорт предшественников аминов клетками новообразования и усиленный гликолитический метаболизм в опухолевых клетках [19].

Позитронно-эмиссионная томография с ⁶⁸Ga-меченными аналогами соматостатина, в настоящее время представляют собой перспективное направление в диагностике нейроэндокринных опухолей. Благодаря фармакологическим характеристикам радиофармпрепаратов и техническим преимуществам метода ПЭТ-КТ, включающим высокое пространственное разрешение и чувствительность детекции, стало возможным визуализировать очаги малого размера, а также образования с умеренной плотностью рецепторов соматостатина. Данное обстоятельство существенно повышает чувствительность и диагностическую точность метода [19].

На сегодняшний день пациентам с НЭО для оценки рецепторного статуса и уточнения распространенности процесса (особенно при высокодифференцированных и умереннодифференццированных НЭО) рекомендуется проводить ПЭТ-КТ с Ga-DOTA-TOC, DOTA-NOC, DOTA-TATE, что также позволяет определиться с возможностью терапии аналогами соматостатина или таргетной радионуклидной терапии [17]. В практике врачи используют данные радиофармпрепараты для топической диагностики опухолей, продуцирующих соматолиберин, АКТГ-эктопированного синдрома, а также для мезенхимальных опухолей, продуцирующих фактор-роста фибробластов 23 [20][21].

Однако диагностическая интерпретация ПЭТ-исследования с аналогами соматостатина сопряжена с рядом диагностических препятствий. К наиболее распространенным из них относится гипердиагностика нейроэндокринных опухолей при выявлении физиологического захвата РФП в области головки поджелудочной железы и крючковидного отростка. Кроме того, очаговое накопление в структурах селезеночной ткани (добавочная селезенка, интрапанкреатическая селезенка, спленоз) может быть ошибочно расценено как метастатическое поражение. Ложноположительные результаты также возникают при интерпретации слабого или умеренного воспалительного накопления, например в лимфоузлах или суставах на фоне остеоартрита, как проявлений метастатического поражения. Для верификации находок необходима обязательная корреляция с анатомическими изображениями, полученными при КТ или МРТ в рамках гибридного исследования, что в большинстве случаев позволяет установить верный диагноз. Окончательная оценка также требует проведения дифференциального диагноза с другими соматостатин-позитивными новообразованиями, такими как менингиома, опухоли нервного гребня и почечно-клеточная карцинома [19].

Радиофармпрепарат ¹⁸F-FDOPA (18F-фтор-дигидроксифенилаланин) также находит успешное применение в оценке функциональной активности и визуализации НЭО. Его механизм действия основан на натрий-независимом транспорте в клетку с последующей декарбоксилированием до ¹⁸F-дофамина, который накапливается в нейросекреторных гранулах [22].

Чувствительность ¹⁸F-FDOPA ПЭТ/КТ является ограниченной (до 25%) для высокозлокачественных НЭО, а также для опухолей, происходящих из передних и задних отделов кишечника. В то же время метод демонстрирует высокую диагностическую эффективность в отношении низкозлокачественных НЭО подвздошной кишки, что объясняется повышенной активностью декарбоксилазы ароматических L-аминокислот — ключевого фермента в биосинтезе серотонина в данных опухолях. В этой подгруппе пациентов ¹⁸F-FDOPA ПЭТ/КТ представляет ценность для точной локализации первичного очага, определения стадии заболевания, а также в выявлении метастатического поражения органов [23][24].

Ограничения применения РФП ¹⁸F-FDOPA связаны с интенсивным физиологическим накоплением препарата в экзокринной паренхиме поджелудочной железы, в связи с чем требуется дополнительная предмедикация карбидопой — периферическим ингибитором декарбоксилазы ароматических L-аминокислот [19].

В рамках эндокринологии использование ¹⁸F-FDOPA ПЭТ/КТ зарекомендовало себя для диагностики медуллярного рака щитовидной железы, параганглиомы, феохромоцитомы, нейроэндокринных образований [17].

Учитывая наличие высокого уровня поглощения глюкозы и гликолиза в раковых клетках, в клинической практике широко с целью топической диагностики используется технология позитронно-эмиссионной томографии с ¹⁸фтор-2-дезокси-D-глюкозой (¹⁸F-ФДГ) в качестве диагностического способа выявления опухолей, определения их активности и определения прогноза заболевания [11]. Принцип метода основан на том, что после транспорта в клетку посредством глюкозных транспортеров (преимущественно типов 1 и 3) ¹⁸F-ФДГ подвергается фосфорилированию под действием гексокиназы. В отличие от глюкозы, образовавшийся ¹⁸F-ФДГ-6-фосфат не метаболизируется, а накапливается внутри клетки, что позволяет проводить его детекцию [19].

¹⁸F-ФДГ ПЭТ/КТ признана методом выбора для визуализации низкодифференцированных НЭО (G3), а также высокоагрессивных опухолей градации G2. Клиническая целесообразность ее применения при новообразованиях G1 и низкоагрессивных опухолях G2 остается предметом дискуссий. При хорошо дифференцированных НЭО G2 пороговым значением индекса пролиферативной активности Ki-67 для целесообразности проведения исследования часто считается уровень ≥10% [25][26].

Интенсивное накопление ¹⁸F-ФДГ ассоциировано с агрессивным фенотипом опухоли и является предиктором неблагоприятного исхода, что подтверждает корреляцию между повышенным уровнем гликолиза и худшим прогнозом заболевания [19].

Однако глюкоза служит основным энергетическим субстратом для многих тканей, поэтому активность ¹⁸F-ФДГ может наблюдаться как в физиологических, так и в доброкачественных состояниях (ткани головного мозга, мочевыделительная система, печень и селезенка, нейтрофилы и активированные макрофаги, в очаге инфекции или воспаления активно накапливают ФДГ). Кроме того, не все опухоли поглощают ФДГ (головной мозг, предстательная железа, нейроэндокринные опухоли) [27].

Согласно данным современных клинических рекомендаций, ПЭТ-КТ с ¹⁸F-ФДГ рекомендуется использовать в качестве метода неспецифической метаболической визуализации при подозрении на низкодифференцированные НЭО или при отсутствии более специфических радиофармпрепаратов. Успешное применение метода описано для диагностики феохромоцитомы, параганглиомы, нейробластомы [17].

Таким образом, выбор способа функциональной визуализации может зависеть от клинической картины заболевания, от предположительного места нахождения опухоли, степени злокачественности и доступности радиофармпрепаратов.

Другим направлением функциональных исследований, которые основаны на оценке метаболических параметров тканей является метаболическое профилирование или метаболомика. Метаболомика предполагает комплексную детекцию низкомолекулярных соединений, формирует новое направление в биомедицинских исследованиях, обеспечивающее интеграцию данных о генетических, эпигенетических и фенотипических характеристиках новообразований [28].

В области метаболомики наиболее часто используются методы ядерного магнитного резонанса (ЯМР) и масс-спектрометрии (МС).

Современные методические подходы включают применение высокоразрешающей ядерно-магнитно-резонансной-спектроскопии с вращением под углом (ЯМР-спектроскопии), позволяющей проводить ex vivo анализ нативных тканевых образцов. Ключевыми преимуществами технологии являются: минимальная потребность в пробоподготовке, высокая степень воспроизводимости, экономическая эффективность, а также доступ к аннотированным базам метаболитов [28][29]. Однако методика требует более высоких концентраций метаболитов, что сказывается на ее чувствительности [1].

Пространственная метаболомика, использующая метод масс-спектрометрической визуализации, позволяет достигать субклеточного разрешения (до 2 мкм), обеспечивая детальную характеристику пространственного распределения метаболитов в тканях. Однако применение данного подхода ограничено исследованиями ex vivo, а технические сложности, связанные с криостатной нарезкой липофильных и минерализованных тканей (жировой и костной), могут снижать точность и воспроизводимость анализа [1].

В пилотном проспективном исследовании Kinross et al. в 2013 г. впервые был применен метод ЯМР-спектроскопии для анализа метаболомного профиля мочи у когорты из 28 пациентов с гастроэнтеропанкреатическими нейроэндокринными опухолями (ГЭП-НЭО), включая исключительно серозно-интрамуральные новообразования. Результаты исследования идентифицировали потенциальные биомаркеры опухолевого процесса [30].

Последующие работы продемонстрировали гетерогенность метаболических фенотипов ГЭП-НЭО, ассоциированную с первичной локализацией (тонкая кишка, поджелудочная железа) и функциональным статусом опухоли. Позднее углубленный анализ 46 образцов тканей НЭО тонкой кишки методами ЯМР-спектроскопии выявил специфические метаболомные изменения, отражающие активацию сложных метаболических путей, потенциально определяющих опухолевую прогрессию и клинические исходы. Характерными метаболическими особенностями солидных НЭО с агрессивным течением являлись: снижение концентраций глюкозы, серина и глицина на фоне повышенного уровня холинсодержащих соединений, таурина, лактата и аланина. Данный профиль отражает адаптацию энергетического метаболизма опухолевых клеток к условиям микроокружения и повышенным биосинтетическим потребностям [28].

Выявление специфического метаболомного профиля опухолевых образований потенциально может использоваться в качестве прогностического биомаркера для корректной стратификации пациентов и выбора подхода к лечению.

Salvia и соавт. опубликовали результаты метаболического профиля пациентов с нейроэндокринными опухолями (НЭО) (77 пациентов с внепанкреатическими НЭО G1-2 и 68 человек контрольной группы). Авторы провели анализ образцов плазмы крови пациентов при помощи газовой хроматографии — масс-спектрометрии (ГХ-МС), капиллярного электрофореза — масс-спектрометрии (КЭ-МС), жидкостной хроматографии — масс-спектрометрии (ЖХ-МС). Было выявлено 34 метаболита, достоверно влияющих на выживаемость пациентов, при этом 10 из них связаны с циклом трикарбоновых кислот. В исследовании были зафиксированы более высокие уровни глутамина, глюкозы и жирных кислот, которые ассоциировались с меньшей выживаемостью пациентов. Также были выявлены усиленные пути метаболизма альфа-линоленовой и линолевой кислот, порфирина, метионина и триптофана [31].

Метаболомика на основе газовой хроматографии и масс-спектрометрии (ГХ-МС) идеально подходит для идентификации и количественного определения низкомолекулярных метаболитов (<650 Да), в том числе малых кислот, спиртов, гидроксикислот, аминокислот, сахаров, жирных кислот, стеролов, катехоламинов, лекарственных препаратов и токсинов. Но часто для того, чтобы сделать эти соединения достаточно летучими для газовой хроматографии, используется химическая дериватизации экстрактов метаболитов, что делает процесс очень сложным и непрактичным для клинических лабораторий [32].

В свою очередь высокоэффективная жидкостная хроматография, обладая преимуществами в виде высокой пропускной способности, специфичности и чувствительности, становится все более популярной для анализа метаболитов энергообмена (лактата, пирувата, НАДН, аминокислот, лактатдегидрогеназы) в биологических образцах, таких как слюна, плазма и моча [1].

Последние достижения показали, что проблемы, связанные с мониторингом метаболизма живых клеток, можно решить в рамках молекулярной диагностики с помощью генетически кодируемых флуоресцентных датчиков, которые связываются с определенными метаболитами, такими как АТФ, и реагируют на них или окисленный и восстановленный никотинамидадениндинуклеотидфосфат (НАДФ(Н)). Эти сенсоры образуют собственные флуорофоры in vivo, как правило, способны избирательно воздействовать на клетки или субклеточные органеллы и обеспечивают точный пространственно-временной мониторинг метаболитов [33]. Zhao и коллеги разработали серию сверхчувствительных, логометрических, генетически закодированных индикаторов лактата, получивших название FiLa (флуоресцентные индикаторы лактата) [1]. В своей работе авторы при помощи датчиков FiLa продемонстрировали накопление лактата в значительной степени в митохондриях и указали, что лактат является ключевым звеном, реагирующим на различные метаболические процессы [33]. Исходно авторы применили технологию для быстрой диагностики сахарного диабета, однако данная технология позволяет определять концентрации не только лактата, но и GLUT, НАДН и НАД, АТФ и имеет перспективы использования и для диагностики опухолевого процесса.

Для регистрации физико-химические процессов, а именно теплового потока, используется метод калориметрии. Этапы диагностики состоят из улавливания теплового потока специальным датчиком, преобразования его в электрический сигнал с последующим анализом [34]. Важнейшим преимуществом изотермической калориметрии выступает возможность количественной оценки термодинамических и кинетических характеристик в режиме реального времени. Такой динамический мониторинг обеспечивает непрерывное наблюдение за метаболической активностью, позволяя регистрировать даже незначительные ее изменения на различных стадиях канцерогенеза или в ответ на применение терапевтических вмешательств [34].

Универсальность технологии открывает широкую область применения метода: анализ кинетических параметров ферментативных реакций, изучение специфичности молекулярных взаимодействий «белок-лиганд», оценки характеристик связывания биологически активных соединений при изучении влияния лекарственного препарата на опухолевые клетки, мониторинг термодинамики фазовых превращений и процессов самоорганизации макромолекул [34].

Несмотря на свои преимущества, изотермическая калориметрия обладает рядом существенных ограничений при изучении метаболизма злокачественных новообразований. К числу основных недостатков метода относятся: низкая пропускная способность при анализе опухолевых клеток, присутствие неспецифических артефактов в многоканальных системах, а также относительно низкая скорость измерений, не всегда соответствующая динамике метаболических процессов в раковых клетках. Дополнительным фактором, ограничивающим применение метода, остается недостаточная изученность точных кинетических параметров теплового потока, характерных для метаболизма злокачественных клеток [34].

На сегодняшний день калориметрия нашла свое применение для оценки уровня следующих метаболитов: лактат, пируват, НАДН и НАД, аминокислоты, лактатдегидрогеназа [1].

Таким образом, все методы оценки метаболизма имеют свои ограничения по чувствительности и специфичности, остаются достаточно трудоемкими, а также имеют ограничения по возможности искажения результатов и влиянию на интерпретацию вследствие пересечения различных типов клеток и метаболических путей [1]. Вместе с тем, развитие и внедрение этих методов представляется наиболее точными трансляционными технологиями будущей медицины, позволяющими оценивать нарушения метаболизма на клеточном уровне и открывающим новые перспективы как в диагностике, так и возможно в лечении.

## ЗАКЛЮЧЕНИЕ

Регуляция процессов энергетического метаболизма представляет собой фундаментальный элемент обеспечения физиологического функционирования клеточных популяций. В условиях онкологического процесса ключевыми проявлениями метаболического перепрограммирования являются интенсификация аэробного гликолиза (эффекта Варбурга), активация глутаминолиза и метаболизма жирных кислот, а также рекрутирование механизмов аутофагии для обеспечения энергетических и пластических потребностей опухолевой клетки. Существующие особенности метаболизма в нейроэндокринных опухолях, такие как преобладание глутаминолиза в митохондриях вместо гликолиза, выступают препятствием для использования стандартных методов топической диагностики. Многообещающим направлением выступает оценка промежуточных продуктов метаболизма гликолиза и окислительного фосфорилирования при помощи ядерной магнитно-резонансной спектроскопии, масс-спектрометрии и исследовании метаболического профиля. Несмотря на существующий методологический арсенал для оценки состояния энергообмена, в настоящее время отсутствует унифицированный подход, обладающий высокой чувствительностью и специфичностью в отношении мониторинга отдельных этапов синтеза аденозинтрифосфата, что актуализирует необходимость разработок по комбинации этих методов в особенности для диагностики и мониторинга опухолей с плохой анатомической визуализацией, а также целого ряда заболеваний, характеризующихся нарушением метаболизма.

## ДОПОЛНИТЕЛЬНАЯ ИНФОРМАЦИЯ

Источники финансирования. Работа выполнена в рамках гранта РНФ №24-15-00283.

Конфликт интересов. Авторы декларируют отсутствие явных и потенциальных конфликтов интересов, связанных с содержанием настоящей статьи.

Участие авторов. Все авторы одобрили финальную версию статьи перед публикацией, выразили согласие нести ответственность за все аспекты работы, подразумевающую надлежащее изучение и решение вопросов, связанных с точностью или добросовестностью любой части работы.
